# Directional responses following recombinant cytokine stimulation of rainbow trout (*Oncorhynchus mykiss*) RTS-11 macrophage cells as revealed by transcriptome profiling

**DOI:** 10.1186/1471-2164-8-150

**Published:** 2007-06-07

**Authors:** Samuel AM Martin, Jun Zou, Dominic F Houlihan, Christopher J Secombes

**Affiliations:** 1 Scottish Fish Immunology Research Centre, School of Biological Sciences, University of Aberdeen, Tillydrone Avenue, Aberdeen, AB24 2TZ, UK

## Abstract

**Background:**

The early stages of the immune response are regulated by key cytokines including both interleukin 1β (IL-1β) and interferon-γ (IFN-γ) which stimulate panels of responsive genes via conserved signal transduction pathways. To further our understanding of the transcriptional response to these cytokines in lower vertebrates we have utilized microarray analysis to characterize the transcriptional response to recombinant rainbow trout IL-1β and IFN-γ in the trout macrophage cell line RTS-11.

**Results:**

RNA was extracted from stimulated or control cells following 6 h incubation and used to hybridize to a salmonid cDNA microarray containing 16,006 different genes. Analysis of the arrays revealed mRNA transcripts that were differentially expressed as a result of exposure to the recombinant proteins, with some responses common for both cytokines. In general the recombinant IL-1β elicited a response where genes involved in the acute phase response were up-regulated, whilst the recombinant IFN-γ induced strong up-regulation of genes involved in the MHC class I antigen presentation pathway. Key genes were chosen that were differentially regulated and analysed by real time PCR at additional time points, up to 48 h following stimulation. This allowed a deeper insight into the kinetics of the response to the cytokines in this cell line.

**Conclusion:**

We demonstrated that in fish both rIL-1β and rIFN-γ stimulated discrete panels of mRNA transcripts which indicted the cells were being directed towards different cellular functions, with IL-β inducing genes involved in the inflammatory response, whereas IFN-γ induced genes associated with antigen presentation.

## Background

The sequencing of several fish genomes including pufferfish [1], *Tetraodon *[2], zebrafish [3], medaka and stickleback (current status reviewed in [4]) has had a tremendous impact on gene discovery within fish. One example is within the immune system, where until recently very few cytokine genes had been discovered using homology cloning or EST analyses [5], but now a large number are known from a wide variety of species including trout, salmon, carp and zebrafish amongst others, and including many interleukins e.g. IL-1 [6,7], IL-2 [8], IL-6 [9], IL-8 [10], IL-10 [11], IL-11 [12], type 1 and type 2 interferons [13-15], lymphotoxin β [16], transforming growth factors [17], chemokines [18,19], as well as some with no obvious homology to known mammalian cytokines. Whilst it is possible to assume the function of cytokines with clear homology to know genes, this is not possible when homology is lacking, and in such cases empirical data is needed on bioactivity. Even when homology is apparent, bioactivity should be confirmed in fish where significant differences in physiology exist.

Advances in functional genomics methodologies now allow the simultaneous measurement of the expression of many thousands of genes, using microarrays. Arrays for fish are becoming increasingly available, and for salmonid fish have been extremely useful in analysing the host transcriptional responses to bacterial [20-23] viral [24], parasitic [25] and fungal infections [26]. Additionally transcriptome analysis has been used to study the response of fish to vaccination [27,28] or stimulation with molecules such as LPS [29]. The microarrays used in the above studies use both cDNA and oligo array type platforms.

To date very few fish cytokines have been produced as recombinant proteins for bioactivity testing. The main exceptions are interleukin-1β [30], TNFα [31] and the interferons [13,14]. In these cases the examination of their function has been rather narrow with the impact on only one or two genes typically examined. In this study two of these cytokines are studied using functional genomics approaches, to give a broad overview of their effect on a large number of genes, to confirm if their predicted effects hold when examined in this way and to give a framework for future studies using cytokines with no clear homology. The two cytokines used are interferon gamma (IFN-γ) and interleukin-1beta (IL-1β), which are expected to have very different biological activities.

Both IL-1β and IFN-γ are cytokines that are key for the early response of the immune system, and as such are key in understanding how cells are directed. IL-1β is a pro inflammatory cytokine directly stimulating the innate immune system [32] and during later stages of infection has major roles in the activation of T and B cells [33]. It is produced as a precursor molecule that is cleaved to generate a mature peptide. It is only active as the mature molecule and can affect most cells and organ systems. There are two primary cell surface receptors that bind the mature IL-1β, type I and type II. When IL-1β binds to the type I receptor, a complex is formed that binds to the IL-1R accessory protein (IL-1RacP), resulting in high affinity binding [34] and a subsequent cascade of signalling that results in transcription factors binding to target genes initiating or decreasing their expression. This signalling pathway is shared with the Toll like receptor signalling pathway via myeloid-differentiation marker (MyD88) and subsequently nuclear factor-κβ (NF-κβ) and mitogen-activated protein kinases [35]. In contrast the type II receptor does not transduce a signal and acts as a sink for IL-1β and may be regarded as a decoy receptor [36].

IFN-γ was originally identified as an antiviral factor but also has central roles in activation of macrophages, stimulation of antigen presentation through class I and class II major histocompatibility complex (MHC) molecules [37] and regulation of T cell differentiation [38]. IFN-γ is produced by natural killer cells and T lymphocytes in response to IL-12 and IL-18 [39]. In its active form the cytokine binds to the IFN-γ receptors R1 and R2 which activate the intracellular JAK/STAT signal pathway to initiate the expression of a large number of different genes [40]. One effect is to induce macrophages to produce toxic substances, including reactive oxygen intermediates, to kill intracellular bacteria and exert antiviral activities by inducing a number of antiviral proteins such as 2', 5'-oligoadenylate synthetase, dsRNA-dependant protein kinase PKR, guanylate binding protein and adenosine deaminase [40]. IFN-γ can also stimulate expression of other cytokines that activate and induce proliferation of CD4+ cells [41]. Thus IFN-γ makes a major contribution to the Th1 induced cell mediated immune response, mainly controlled by mutual feed back between IFN-γ and IL-12 [42].

The macrophage response to infection and activation by immune stimulants can be effectively analysed by microarray analysis allowing thousands of genes to be monitored for expression in parallel. This can be seen by the number of studies on mammalian (reviewed [43]) and avian [44] macrophage microarray studies. The methods employed by pathogens to evade the immune system are complex and by studying specific cell types or tissues the host defence strategies can be examined. To further characterise the response, specific cytokines have been used to stimulate human [45], murine [46] and bovine [47] macrophage cell lines. In addition the gene expression response of T cells to PAMPS has been explored using microarrays [48].

Recently, both IL-1β [6] and IFN-γ [14] have been cloned and sequenced from rainbow trout in our laboratory and recombinant proteins produced to study their respective functions [14,30]. Thus it is possible to study for the first time the genes that are responsive to these two key cytokines on a transcriptome wide scale in a lower vertebrate. Stimulations were performed with a rainbow trout macrophage cell line RTS-11 [49], expected to be highly responsive to both cytokines, as the model used, to keep intra assay variation low and to allow maximal power to the subsequent data analysis. A 16,006 feature salmonid microarray [50] was used to identify genes that were altered in expression as a result of stimulation with these recombinant cytokines, with the prediction that differential gene profiles would be induced that indicate a bias towards innate or adaptive immunity. This platform has previously been demonstrated to be useful in the transcriptional analysis during infection and immune response in salmonid fish [25,26].

## Results

### Cell stimulation

To dissect the immunomodulatory effects of the rainbow trout recombinant proteins on RTS-11 cells, the cells were treated with either 20 ng ml^-1 ^rIL-1β or rIFN-γ protein. Control cells were unstimulated. Prior to microarray analysis rt PCR was performed using genes known to be stimulated by these cytokines with samples taken at 6 h post stimulation. For IL-1β, IL-1β and IL-8 expression were induced, whilst for IFN-γ, γIP [51] was shown to be increased in expression, confirming the cells were responding to the stimulation (data not shown). On the basis of these results the RNA was used to continue with the microarray experiment and time course response. Later time points were not examined by microarray to avoid confounding effects of indirect actions of the two cytokines, with 6 h considered optimal for direct effects of both based on our previous studies (see methods).

### Microarray analysis

The salmonid cDNA microarray developed by von Schalburg et al. [50] that contained 13,421 Atlantic salmon and 2,576 rainbow trout cDNAs was used in this study. Characterization of this microarray [50], demonstrated that rainbow trout cDNA hybridizes well even though the majority of sequences are for Atlantic salmon, because of the very high level of sequence conservation between these two species. A total of 5963 (± 347 SEM) features had a signal that was greater than the background threshold to be included in the working data set.

For the IFN-γ experiment, 34 cDNA features were found to be significantly increased 2 fold or greater and 8 genes down regulated using the same criteria. For cells stimulated with IL-1β, 92 genes were increased and 7 decreased. Fifteen of the genes altered in expression were common between the two cytokine stimulations. Fewer cDNA features were down regulated for cells stimulated with rIFN-γ and rIL-1β, indicating that the cytokine stimulation at 6 h has a predominantly positive effect on gene transcription. The cDNAs that were found to be differentially expressed by the microarray analysis are shown in Table 2.

**Figure 1:**
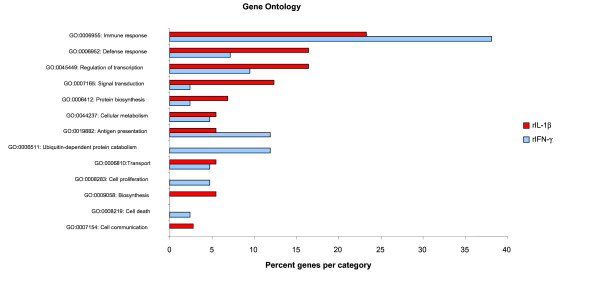
**Numbers of differentially expressed genes grouped by Gene Ontology identifier for Biological process**. Summary of function of proteins encoded by genes found to be up regulated after cytokine stimulation of RTS-11 cells as revealed by microarray analysis. These ontologies are only for those genes for which a functional protein could be assigned; 86% and 61% for rIFN-γ and rIL-1β induced genes respectively. For GO annotation high level annotations were used.

The majority of cDNA identities were assigned on the GAL file [50], however 28% of clones were unidentified. To assign further identity to these unknowns the "Unigene" sets were utilized [52]. For salmon and rainbow trout 21,841 and 23,359 Uni genes have been determined to date (26/09/06). Here ESTs have been clustered and BlastX performed on the longest cDNA in the cluster [53]. The identities that were directly from the UNI gene sets are marked in Table 2. UniGene clusters with no functional protein assigned to them are given the UniGene code which may help in future identification. Genes that do not have a functional protein or a UniGene cluster are termed unknown, indicating they only occur once in the data base. Of the cDNAs that are found to be altered 91% and 71% for rIFN-γ and rIL-1β respectively were found to have identities with functional proteins. All cDNAs that had a functional protein identity had gene ontology identifiers assigned to them and were grouped according to gene ontology function. Gene ontology of up regulated genes are shown in Fig. 1.

Of the four cDNA features from the IFN-γ set with no identified homologue, 3 of these were members of clusters as assigned by NCBI UniGene and one was a singleton that did not match any other sequence in the data bases. For the IL-1β group of the 27 with no protein identity, 19 were in clusters with four found to be singletons.

Fourteen identified genes and one cluster [NCBI:Ssa.15825] were found to be up regulated by both IL-1β and IFN-γ and included a range of encoded proteins. The magnitude of response, in terms of the level of expression of genes affected by both cytokines, was markedly different between the two stimulations. This is clearly seen for the C type lectins which are dramatically stimulated by IL-1β (18 fold) whilst they are increased 6.5 fold by IFN-γ. Several of the key genes involved in the IFN-γ signalling pathway are strongly up-regulated in cells stimulated with IFN-γ, such as IRF-1 (×12 increase) and an IRF-8 homologue (×10 increase). In contrast IRF-1 was only increased 3 fold after IL-1β stimulation. For down regulated genes, one gene was decreased by both cytokines, referred to as ID2 protein. This protein is involved in inhibition of protein binding to DNA and as such could be described as an inhibitor of DNA transcription [54].

### Real time PCR confirmation of expression

**Figure 2:**
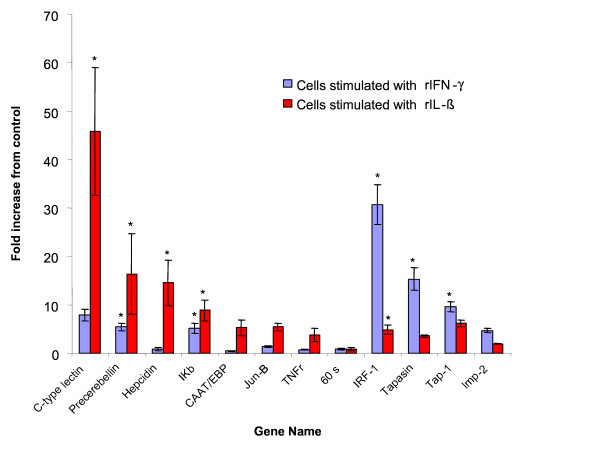
**Real time PCR results on 6 h mRNA samples that had been used for microarray analysis**. Each selected gene was examined for expression following 6 h stimulation with either trout rIL-1β or trout rIFN-γ. The expression of each gene was normalised to β-actin. The real time PCRs were all performed in triplicate and are shown as mean ± SEM. (* indicates the increase in expression compared to control is significant by t test P < 0.05). Genes were ordered in relation to largest fold increase induced by rIL-1β or rIFN-γ with the 60S ribosomal protein not showing induction by either cytokine.

**Figure 3:**
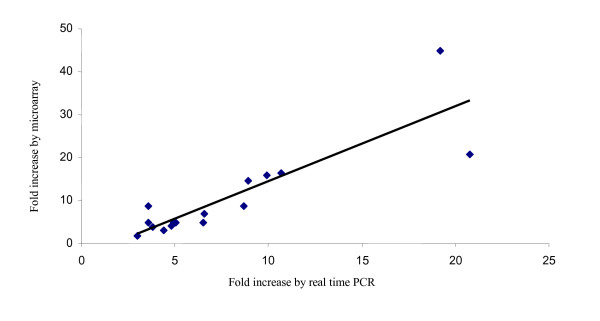
**Analysis of the expression induction by both microarray and real time PCR**. For the microarray data, the mean value was used if the gene occurred more than once on the microarray. There is a significant correlation between the values obtained by microarray and real time PCR (Pearson correlation = 0.864, P < 0.001).

Real time PCR was used to confirm the expression of a subset of genes found to be differentially expressed during the microarray hybridizations at 6 h post stimulation. Initial real time PCR products were separated on 2% agarose gels and viewed under UV to confirm only a single product of the predicted size was being amplified. The primer sets were used for cDNA from both stimulations to add additional information not obtained from the microarray, as some genes were only assigned as differentially expressed by one cytokine. The candidate gene mRNA expression was normalized to both β-actin and to ELF-1α, neither of which were found to vary by microarray analysis. The real time PCR results when normalized with β-actin are shown in Fig. 2. The results obtained when normalized with ELF-1α are very similar and correlate significantly with the data normalized with β-actin (Pearson correlation = 0.998, P < 0.001). To compare the expression pattern obtained for the microarray and the real time PCR data the fold increase was plotted (Fig. 3) and shown to be highly correlated (Pearson correlation = 0.864, P < 0.001) indicating both sets of results generally agree.

All cDNAs used in the real time PCR analysis confirmed the up regulation observed in the microarrays except one cDNA encoding a 60S ribosomal protein L30 (CA037550) which was assigned as up regulated in cells stimulated with IL-1β but was not found to be induced during the real time PCR assays.

### Temporal gene expression analysis by real time PCR

**Figure 4:**
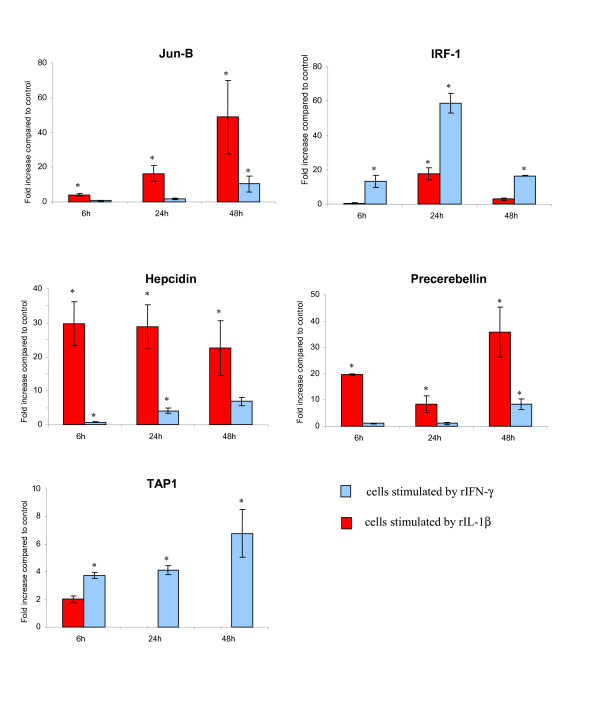
**Temporal expression of genes stimulated by rIL-1β and rIFN-γ in RTS-11 cells**. Cells were incubated for either 6, 24 or 48 h with recombinant cytokine at 20 ngml^-1^. The real time PCRs were normalized against β-actin expression. *Indicates if the expression in the stimulated cells is significantly different than the control cells by t-test (P < 0.05).

A second stimulation on the RTS-11 cells was performed to obtain further time points (6, 24 and 48 h post stimulation) to allow the kinetics of the transcriptional response to be monitored. Five genes were chosen for this kinetics study, the transcription factors Jun-B and IRF-1, precerebellin and hepcidin representing acute phase response genes and TAP1 as a gene involved in antigen presentation. Fig. 4 shows the expression differences obtained for these later time points. When stimulated with rIL-1β Jun B increases throughout the time course to a maximum of 50 fold increase at 48 h compared to the unstimulated control. This gene is also increased by rIFN-γ stimulation but to a much lower extent. The IRF-1 gene has a transient change in expression, being maximal at 24 h, then decreasing by 48 h. This response profile was also observed for the γIP gene following rIFN-γ stimulation, where this gene also showed maximal expression at 24 h [14]. IRF-1 is also stimulated by rIL-1β but to a much reduced level, and interestingly it had the same expression profile as when stimulated with IFN-γ with highest induction at 24 h. Temporal expression of hepcidin by rIL-1β stimulated cells shows maximal expression at 6 h, with expression slowly decreasing to 48 h, although still being highly induced (25 fold greater expression than in nonstimulated cells). The opposite is true of hepcidin mRNA expression following rIFN-γ administration, where there is a steady increase in gene expression during the time course. One gene representing the antigen presentation pathway, TAP1 was also monitored, and also continued to increase during the time course under rIFN-γ stimulation, suggesting an increasing capacity for MCH class I presentation. There was also an increase in expression in cells stimulated with the rIL-1β at 6 h as observed in the microarray, but following this no signal was detected indicating this was only a transient increase.

## Discussion

In vertebrates, innate immunity provides a first line of host defense against pathogens and the signals that are needed for the activation of adaptive immunity. Activation of the endogenous inflammatory response by both IL-1β and IFN-γ will give key insights to the mechanisms by which these cytokines function in fish. We hypothesized that stimulation with these recombinant proteins may identify genes that could help develop models for analyzing the progression of the inflammatory response in lower vertebrates. Studies with cultured cells avoids the complex host and cell type response observed using in vivo experiments and the application of DNA microarray technology is expected to reveal many of the cytokine responsive genes

Recently we have produced biologically active recombinant fish cytokines IL-1β and IFN-γ from rainbow trout [14,30]. To further this work we have stimulated a macrophage cell line (RTS-11) with these recombinant proteins and characterized the transcriptional response. Classically IL-1β is stimulated early during an infection or stimulation of cells, with 3–6 h being an optimal time for IL-1β to be expressed and then to induce down stream genes [55]. IFN-γ provokes a large variety of immune responses by stimulating intracellular gene expression. In particular it is a potent activator of macrophages, and in addition to this it increases antigen presentation and attracts leukocytes to infected tissue sites, mainly through induction of gene expression of a number of factors including MHC molecules and chemokines such as γ-IP, a T cell attractant [56].

**Figure 5:**
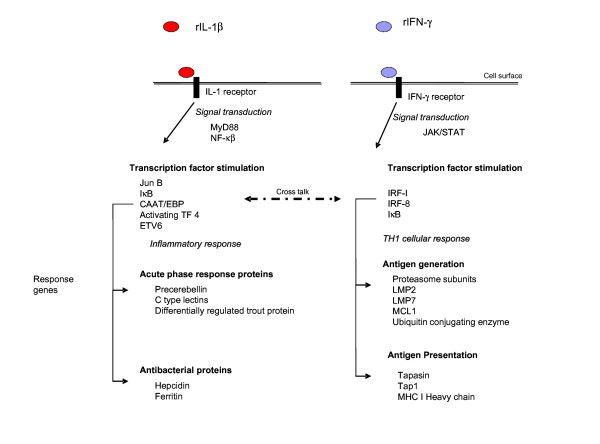
**Proposed model for transcriptional responses in RTS 11 cells**. Key transcriptional differences observed following stimulation of rainbow trout RTS 11 cell line with recombinant IL-1β or IFN-γ are shown.

The micro array analysis performed here has demonstrated there are a variety of genes induced following stimulation with these two recombinant proteins, with a number induced by both cytokines, although the scale of induction varies. In general terms we show that trout rIL-1β induces transcription of genes that may bias the cells towards an acute phase type of response, with key genes stimulated having this function, whilst rIFN-γ appears to be inducing genes indicative of an adaptive Th1 response. A model diagram highlighting these key features is shown in Fig. 5. A large proportion of the genes stimulated by trout rIL-1β are involved in antibacterial activities or activation of antibacterial responses, with many of these genes being characterised previously as acute phase response proteins. Although the acute phase response proteins are usually found in liver, in fish they are expressed in many immune responsive tissues [20] and in this study in a macrophage cell line. Central to the coordinated response is the activation and production of nuclear transcription factors. rIL-1β stimulates transcription factors including Jun-B, C/EBP, and IκB which transduce the signal from the cytokine IL-1 receptor to responsive genes. A key antibacterial peptide, hepcidin is strongly induced by rIL-1β. This gene is stimulated in a variety of fish species in response to various bacterial pathogens, as seen in Atlantic salmon [20,57], winter flounder [58] and hybrid stripped bass [59], and in mammals several inflammatory cytokines including IL-1β [60] and IL-6 [61] can regulate hepcidin production. Probably this expression is an indirect affect of IL-1β, through its ability to induce gene expression of transcription factors that bind to the promoter of the hepcidin gene. Both NFκB and C/EBPβ sites have been found in the promoters of mammal [62] and fish hepcidin genes [57,63], and in this study it was observed that C/EBP is increased by rIL-1β. The precise method by which bacteria are killed by hepcidin remains unclear, but may be linked to hepcidin being a negative regulator of iron metabolism [64]. It appears in fish that the key role of hepcidin is as an antimicrobial peptide, but through evolution it has also become an iron regulatory hormone; the extent of iron regulatory function of hepcidin in fish is still unknown [65]. A major iron regulator, ferritin (also considered an acute phase response protein) was also up regulated by rIL-1β, and functions to prevent the proliferation of an invading bacterial pathogen. This primitive yet effective antimicrobial mechanism operates by depriving microbial organisms of their nutrients in what is commonly referred to as the iron-withholding strategy of innate immunity [66]. This up regulation of ferritin mRNA is increased following differing bacterial infections in both vertebrates and invertebrates such as shrimp [67]. In mammals this expression is regulated by pro inflammatory cytokines at both the transcriptional and translational level [68], however the mechanisms in lower vertebrates and invertebrates are yet to be determined.

Correct pathogen identification is essential in order to elicit a protective host response, which can occur through recognition of PAMPS. Lectin receptors function to bind sugar motifs on the surface of invading pathogens and in activation of complement and as such are important in the innate defence against bacterial infections. This mechanism is conserved throughout vertebrate evolution [68]. Lectin and Toll receptors have related functions, both recognising conserved products of microbial metabolism [70]. C-type lectin mRNAs are widely reported to be increased following different bacterial infections in salmonid fish such as *Piscirickettsia salmonis *[22], *Aeromonas salmonicida *[20,21,57], and by infection with *Vibrio anguillarum *in rainbow trout [71]. Lectins may be involved in signalling following virus infection as seen with Rhabdovirus infection [72] and can be induced by administration of the double stranded RNA poly I:C [73]. In this study C-type lectins were highly induced by rIL-1β and to a lesser extent by rIFN-γ, indicating there is an increase in production during the inflammatory response to increase recognition of pathogens and activate the lectin pathway of complement. Evidence is growing that lectin like receptors can synergise or antagonise toll like receptor signalling which may occur via the immuno receptor tyrosine based activation motif (ITAM) found in the TLR2 receptor [74]. In common with other acute phase response proteins, C-type lectins have transcription factor binding sites for NFκB, NF/IL-6 and C/EBP [75]. A galactose binding lectin, galectin, stimulated in this study by rIFN-γ is described as an eosinophil chemoattractant produced by activated T lymphocytes. Endothelium cells from humans suffering from inflammatory disease express galectin-9, known to be stimulated by IFN-γ and as such, galectin may help regulate interactions between the vascular wall and eosinophils during the inflammatory response [76].

A factors involved in cell differentiation and commitment is a myristoylated alanine-rich C kinase (MARCKS) found increased by IL-1β. MARCKS also has a role in macrophage and neutrophil motility and functions to direct cells towards proliferation [77]. In addition ETV-6 is a transcription factor (Ets factor) increased by rIL-1β that results in differentiation and commitment of a cell to a specific fate. Ets factors play a critical role in the regulation of genes involved in tissue-development, differentiation, cell cycle control, and cell proliferation as both transcriptional enhancers or repressors, some of which are located in the MHC cluster [78]. This could indicate rIL-1β is initiating the reprogramming of the RTS-11 cells.

IFN-γ exerts its effect via a signalling cascade through the Jak/Stat pathway [79] which stimulates interferon response factors which either enhance or inhibit expression of interferon responsive genes. Two interferon response factors (IRF) 1 and 8 are dramatically up regulated by rIFN-γ stimulation demonstrating the cells are responding to the IFN-γ in a predictable manner. A major group of genes that are stimulated by IFN-γ are those involved in MHC class I antigen presentation [37]. MHC class I molecules present endogenously synthesized peptides including virus peptides following viral infection. Upon stimulation with IFN-γ many of the genes in this pathway are transcriptionally stimulated and the cell can then increase the rate at which these peptides are presented on the cell surface for subsequent recognition by cytotoxic T cells [80]. This pathway involves proteins being targeted by ubiquitination and directed to the proteasome which digests the protein into peptides that are bound to MHC class I and β2 microglobulin before being transported to the cell surface. Once the proteins have been cleaved into peptides by the proteasome they are transported to the endoplasmic reticulum and the TAP and tapasin molecules bind the peptides to the MHC class I heavy chain and in the presence of β2 microglobulin form the mature MHC molecule that is presented on the cell surface. Three subunits from the proteasome LMP2, LMP7 and MELC1 as well as TAP 1 and tapasin mRNAs were significantly increased in expression in the IFN-γ stimulated cells. β2 microglobulin was also increased but was not included in the results Table 2 as it was less that 2 fold increased. These results clearly indicate the RTS-11 cells are being primed by the recombinant IFN-γ to follow a Th1 pathway to stimulate an adaptive immune response.

There are a number of genes that are stimulated by both rIFN-γ and rIL-1β. These include IκB which is involved in the regulation of NFκB activity. IκB binds to NFκB to inactivate it, but during activation by cytokine stimuli the IκB is phosphorylated by IκB kinase (IKK) and subsequently unbiquitinated and destroyed by the proteasome [81]. Several genes encoding the proteins involved in antigen presentation are also transcriptionally increased by rIL-β, but at a lower level than with rIFN-γ stimulation, indicating that this is not a major function of IL1-β. rIL-1β also increased IRF-1 expression but to a much lower degree than found with rIFN-γ stimulation. Together these results demonstrate there is cross talk between the regulation of genes controlled by these two cytokines, with a possible co stimulatory role via IL-12 mediated induction of IFN-γ [82] and this may be the mechanism by which the cytokines stimulate the same subset of genes. Three mRNAs are stimulated by rIL-1β for which the precise functions remain unknown. These are precerebellin [71], "Differentially regulated trout protein" [71], and "Immune response protein 1" [22], the latter is stimulated by both rIL-1β and rIFN-γ. These genes may be regarded as markers of a major immune function responding in a positive manner to inflammatory stimuli and are likely to be regulated in a similar manor to acute phase response proteins.

The expression difference of ten mRNAs were chosen for extended analysis by real time PCR to determine if the difference found by microarray analysis could be confirmed by real time PCR. These genes showed exclusively or preferentially increased expression by one or other of the cytokines and represented the major functional groups found to be stimulated by the microarray analysis. Hepcidin, C type lectin and precerebellin were chosen to represent acute phase response proteins and antibacterial peptides. Genes encoding transcription factors JunB, C/EBP, IκB and IRF-1 were selected as these may be central to progression of the cells response and genes representing antigen presentation included TAP1, LMP2 and tapasin. Two further genes, a tumor necrosis factor (TNF) receptor which represents a proinflammatory cytokine receptor and a 60s ribosomal protein mRNA as an indicator of cellular protein translation, were also used. The expression profile from the microarrays correlated well with the results obtained from the real time PCR indicating robust analysis of the microarray. The highly significant correlation when the real time PCRs were normalised to either β-actin or ELF-1α shows that both these control genes are suitable for normalizing the IL-1β and IFN-γ responsive genes which are changed in expression.

The real time PCRs investigating the temporal expression revealed that there is a limited amount of co regulation of genes responding to both rIL-1β and IFN-γ as discussed earlier. Interestingly, several of the genes respond to the stimulants with different kinetics. The antibacterial peptide hepcidin and precerebellin are expressed at very low levels 6 h after IFN-γ stimulation, but the expression continues to rise until 48 h, this could be as a result of the IFN-γ stimulating genes which in turn are activating NFκB signal transduction pathway and causing this later expression. Although this is a much lower fold difference than seen with rIL-1β it may indicate the acute phase response is retarded when cells are stimulated with rIFN-γ compared to rIL-1β. Conversely TAP1, which may indicate a shift towards adaptive immunity, increases in expression throughout the time course after IFN-γ stimulation. An increase is observed at 6 h after IL-1β stimulation but then no expression difference is observed at 24 and 48 h indicating this pathway is not increased over time by IL-1β. There may be an autocrine effect following the stimulation with IL-1β that affects the expression of precerebellin, since the gene expression decreases at 24 h and then rises again at 48 h relative to the control. This may reflect increased IL-1β expression by the RTS 11 cells themselves that causes a secondary enhanced response at 48 h.

At the most basic level all vertebrates need to respond in the correct manner to pathogens, with control of gene expression during the response being central for successful elimination of the pathogen. Throughout the vertebrates there are common sets of genes which are activated during this response although many pathogens attempt to disrupt this response, often by altering the effect and profile of cytokines. To remove pathogen and host effects we have used a cell line to directly study the effect of cytokines on the transcriptional response, and our results obtained here can be directly compared to transcriptome changes observed in mammalian cells following cytokine stimulation. IL-1β stimulation increases transcription for genes encoding proteins associated with the antipathogen response, proliferation and inflammation, via the NFκB signal transduction route [82,83]. For IFN-γ stimulation in various mammalian cells, gene sets similar to those found in fish show modulated expression, with genes encoding proteins related to antigen presentation and immunomodulatory activities stimulated in both macrophages [47,84] and astrocytes [85]. From this we can speculate that the general response to these key cytokines is conserved throughout vertebrates although there can be variation in response depending on factors such as cell type and incubation time [43].

## Conclusion

In conclusion, these results further our understanding of gene expression alterations induced by specific cytokines in salmonid cells. This is the first time that an in depth study has been performed using recombinant cytokine molecules for transcriptional profiling in a lower vertebrate. We clearly demonstrate that the rIL-1β enhances the expression of molecules involved in the acute phase response and innate immune mechanisms, whereas a major shift in gene expression towards the adaptive response and MHC class I antigen presentation is seen when using rIFN-γ. Gene ontology confirmed that the major groups of genes altered in expression were related to the immune response.

## Methods

### Cell culture

The rainbow trout macrophage cell line RTS-11 [49] was maintained in L15 medium containing 30% FCS (Labtech International) and antibiotics (100 μg ml^-1 ^penicillin and 100 U ml^-1 ^streptomycin, P/S) at 20°C. Cells were passaged to fresh flasks at 80% confluence and cultured for 2 days before stimulation with the recombinant proteins for 6 h at a concentration of 20 ng ml^-1^. This time point and concentration was chosen on evidence from previous experiments, with rIL-1β stimulated genes including cycloxygenase-2 and IL-1β being induced maximally at 6 h [30] and rIFN-γ also showing good stimulation between 4 and 8 h of genes including guanylate-binding protein [86] and γ-IP being maximally stimulated at 6 h [13]. Later timings also have the potential issue of indirect effects caused by induced gene products. For both recombinant cytokines the response of stimulated genes plateaus with a concentration of between 10 and 20 ng ml^-1^, with higher concentrations not giving significantly higher transcription levels [14,30,86]. Cells were cultured in 80 cm^2 ^flasks for the microarray experiment but for the time course experiment cells were cultured in 25 cm^2 ^flasks for 6, 24 or 48 h.

### RNA isolation

The RTS-11 cell line is a mixture of adherent and non adherent cells. The non adherent cells were pelleted by centrifugation at 600 g for 5 min. These pelleted cells were lysed and combined with the adherent cells. Total RNA was extracted using the RNA STAT60 reagent (AMS Biotechnology) according to the manufacturer's instructions. RNA was resuspended in DEPC treated water and the concentration and quality of the RNA was determined by spectrophotometry and using an Agilent Bioanalyser 2100 (for RNA integrity).

### Microarray hybridization

The microarray used in this study was constructed from cDNAs selected from 18 high complexity salmonid cDNA libraries and consisted of 16,006 cDNA features [50]. The majority of cDNAs selected for the chip came from a normalized mixed tissue library (spleen, kidney and brain). The full list of genes contained on this array can be found at . For array hybridizations triplicate RNA samples were used that were obtained from RTS-11 cells stimulated with either trout rIL-1β or rIFN-γ, as described earlier or unstimulated control cells. Next, 20 μg total RNA was primed with oligo dT primer and reverse transcribed in the presence of amino allyl modified dUTP following the manufacturer's instructions (Post Labelling Kit, Amersham Biosciences). Following reverse transcription, RNA template was removed by sodium hydroxide treatment and unincorporated nucleotides removed using a spin column (GFX spin Column, Amersham Biosciences). The fluorescent label, either Cy3 or Cy5 was added to the purified cDNA in the dark in a volume of 30 μl and the labelled cDNA purified from free dye on a GFX column. For the dye swap experiments the cDNA was split to two equal aliquots before the dye was coupled to the cDNA. Six slides were used in total per experiment, i.e. for each recombinant cytokine. Prior to hybridization 1.5 μl d(A)_80 _(1 mg ml^-1^), 110 μl formamide, 55 μl hybridization buffer (Amersham Biosciences) and water was added to give a final volume of 220 μl. The cDNA was heated to 95°C for 2 min before being added to the hybridization chamber. The hybridization was performed for 16 h at 42°C on an Amersham Lucidea hybridization station. Prior to hybridization slides were pretreated by heating in water at 95°C for 5 min, then washed with isopropanol and air dried. Post hybridization washes (10 min each) were as follows: 1× SSC (45°C), 1× SSC +0.2% SDS (45°C), 0.1 × SSC + 0.2% SDS (20°C). Slides were then dried before being scanned.

### Microarray image analysis

After hybridization and washing, the slides were scanned on an Axon 4200A scanner (Axon Instruments) at a resolution of 10 μm and saved as *.TIF files. The detected flourescence was adjusted by altering the photo multiplying tube (PMT), this ranged from 700–820 (arbitrary units) to give an intensity ratio of the slides of approximately one. Initial image analysis was performed with the GenPix (version 5.1, Axon instruments) programme and the array images edited to ensure that the GAL (Gene Associated List) file was correctly orientated and that any abnormal hybridization signals were flagged as "bad" and not included in subsequent analysis. Edited images were imported into Acuity (version 5.1, Axon instruments) and analysis performed. All arrays were background corrected using local background correction and normalized using Lowess normalization [87]. The data was filtered to remove bad signal and signal that did not reach a predetermined threshold (signal to noise ratio >1.5 for either Cy3 or Cy5). Data sets of genes assigned as up or down regulated were where the gene was significantly different by t test (P < 0.05) and the magnitude of difference was ≥ 2 fold increased or decreased. Gene ontology identifiers were used to group genes encoding proteins by function, using the UniProt [89] and the Gene Ontology Consortium [90] web sites maintained by the European Bioinformatics Institute, using both automatic and manual assignment. All GO identifiers are at high level.

### Real time PCR gene expression analysis

Total RNA was collected from cells that were stimulated as described above for 6, 24 and 48 h. For real time PCR, RNA was denatured (65°C, 10 min) in the presence of 1 μl oligo dT_17 _primer (500 ng μl^-1^), the RNA was cooled on ice and cDNA was synthesised using 15 U Bioscript reverse transcriptase (Bioline, UK) in the presence of dNTPs (final concentration 200 μM each), at 42°C for 1 h in a final volume of 20 μl. The cDNA was diluted 5 fold to 100 μl and 3 μl used as the template for PCR using primers designed against the rainbow trout genes of interest. The chosen clones and their corresponding primers are shown in Table 1. PCR amplification was performed using an Opticon real time PCR machine, using ready prepared 2× master mix sybre green PCR mix (Biorad) with a final PCR volume of 25 μl, in white 96 well plates (Biorad). PCR conditions were 95°C for 5 min followed by 94°C for 20 sec, 57°C for 20 sec and 72°C for 20 sec. The fluorescence signal output was measured and recorded at 78°C during each cycle for all wells for 35 cycles. A negative control (no template) reaction was also performed for each primer pair. A sample from the serial dilution was run on a 2% agarose gel and stained with ethidium bromide and viewed under UV light to confirm a band of the correct size was amplified.

**Table 1:** Primer sequences used for gene expression analysis by real time PCR. Amplification was detected by Sybr Green using an Opticon real time PCR machine (BioRad).

**Gene**	**Accession number**	**Short name**	**Sequence**	**Annealing temp used**	**Product size**
HEPCIDIN	BX868476	Ext1F1	TGCAGTGGTACTCGTCCTTG	58	167
		Ext1R1	GACGCTTGAACCTGAAATGCTCC		
IRF-1	AF332147	IRF-1 F1	CCGCTGTGCAATGAACT	60	285
		IRF-1 R1	AGGCTGTCTGTGCTGTCTACTAT		
C-TYPE LECTIN	DV191936	Ext2tF1	TCTCCTGTCCCATTTTGCTT	58	238
		Ext2R1	GATCCGCCATCCACATATTC		
PRECEREBELLIN	AF192969.2	Ext3F1	GCCTTCTCCGCTTCCTTTAT	58	185
		Ext3R1	TTCCCAACATTGCAAGTGAA		
IN NFKbeta	OMY317969	Exts4F1	GAAGCCACAGAACATGCTGA	58	242
		Exts4R1	TGGGTGATCACACTGAAGGA		
JUN-B	CF752495.1	Ext5F1	TACTGCACTGTTGGGACAGC	58	167
		Ext5R1	CAGAATGCCCCGAGTGTTAT		
LMP2	AF112117.1	Ext6F1	AAGTTCGTTCAGCTGCCACT	58	234
		Ext6R1	GTTGGCAGTCCTCTTTGCTC		
TAP1	AF115536	Ext7F1	CCATGAGTCGCATACACACC	58	188
		Ext7R1	AGTGACCCGCATGAAGTACC		
60S rp L30	CA037550	Ext9F1	GGGATACAAGCAGTCCCAGA	58	211
		Ext9R1	GGGTCGATGATAGCCAGTGT		
CCAAT/EBP	AY144611.1	Exst10F1	CAGCGGGTGTTAAGATCCAT	58	200
		Exst10R1	GCAGCAGGAGGATCCAAGTA		
TNF rec	OMY517804	Ext11F1	CACCGACTGTGGCAAGTCTA	58	227
		Ext11R1	GGGTCACAGGTAGGCAGTGT		
TAPASIN	DQ092322	Ext12F1	GCACGGTGTACTTGCCCTAT	58	188
		Ext12R1	AGGCCCTGTTAACTCCCAGT		
β-ACTIN	AF012125	RTBAEF	CCAGGCATCAGGGAGTGA	60	283
		RTBAER	GTACATGGCAGGGGTGTTGA		
ELF-1α	AF321836.1	EF1AEF	CAAGGATATCCGTCGTGGCA	60	317
		EF1aER	ACAGCGAAACGACCAAGAGG		

**Table 2:** cDNAs identified as differentially regulated by microarray analysis. Clone ID^1 ^is the accession number of the cDNA feature on the microarray, when more than one cDNA feature was the same sequence the accession number for the feature with the highest fold difference is given, the number in brackets indicates the number of features identical and used in analysis. Identity^2 ^is the functional protein assigned to the cDNA feature, Accession^3 ^number relates to the identify of the protein. *indicates cDNA features where protein function is determined from NCBI UniGene as described in the text. Mean is the mean fold difference between the stimulated and non stimulated control cells.

**Clone ID^1^**	**Identity^2^**	**Accession^3^**	**Gene Ontology Identifier**	**Mean**	**SEM**
***Up regulated by rIFN-γ***					
					
CA063565^(2)^	Interferon regulatory factor 1	AAM77843	GO:0003700: transcription factor activity	12.1	4.9
CA062585^(2)^	Tapasin	ABE27285	GO:0019882: antigen presentation	11.4	3.5
CA063956	Complement C1q	XP_544507.2	GO:0006955: immune response	11.4	2.7
CB505764	C1q-like adipose specific protein	AAM73701	GO:0006955: immune response	10.6	5.8
CA062838*^(2)^	Acyl-coenzyme A-binding protein	AJ632152	GO:0006810: transport	10.3	1.4
CA051393*	Interferon consensus sequence binding protein (IRF-8)	NP_990747.1	GO:0003700: transcription factor activity	10.0	4.7
CA062698*	Ssa.15825		N/A	9.6	5.0
CB506130	Proteasome subunit beta type 1-A 20S	O09061	GO:0006511: ubiquitin-dependent protein catabolism	9.2	6.2
CA062737^(3)^	Low molecular mass protein 7	P28063	GO:0006955: immune response	8.8	3.3
CA052500	TAP 1	Q9JJ59	GO:0019882: antigen presentation	8.7	1.4
CA056108^(2)^	C-type lectin 2-1	Q91ZW7	GO:0006955: immune response	8.5	3.8
CA055080	Core histone macroH2A2.2; H2A histone family	AAH76893	GO:0044237 : cellular metabolism	7.6	4.3
CB516220^(2)^	Inhibitor of nuclear factor kappa B alpha	Q9Z1E3	GO:0006955: immune response	7.3	3.9
CA057910*	Programmed cell death 1 ligand 1	XP_424811.1	GO:0008219 : cell death	6.8	1.5
CA051372^(2)^	Low molecular mass protein 2	Q60692	GO:0006955: immune response	6.5	2.5
CB511048	VHSV-induced C-lectin-like protein	AY572832	GO:0006955: immune response	6.1	3.0
CA063009*^(2)^	Ssa.6457		N/A	6.0	1.7
CA052717*	Fc receptor-like 4	XP_547521.2	N/A	5.7	2.0
CA050149	IMMUNE-RESPONSIVE PROTEIN 1	XP_542615	N/A	4.1	1.6
CA050971	Proteasome subunit alpha type 6	Q9QUM9	GO:0006511: ubiquitin-dependent protein catabolism	3.8	1.5
CA063863*	Similar to interferon regulatory factor 1	NP_002189.1	GO:0003700: transcription factor activity	3.6	0.4
CA052613	Ubiquitin conjugating enzyme	XP_517968	GO:0006511: ubiquitin-dependent protein catabolism	3.6	0.6
CB500108	Ribosomal protein L35	AAM34649	GO:0006412: protein biosynthesis	3.4	0.8
CA044026^(2)^	MHC class I	L07605	GO:0019882: antigen presentation	3.4	0.4
CA052774	Protein phosphatase 2	P62715	GO:0044237 : cellular metabolism	3.0	0.5
CB511680^(2)^	Lysozyme	P17897	GO:0006952: defense response	3.0	0.3
CA055186	MECL1	P70195	GO:0006511: ubiquitin-dependent protein catabolism	2.7	0.5
CB493732	Proteasome subunit beta type 7	NP_989728.1	GO:0006511: ubiquitin-dependent protein catabolism	2.7	0.7
CA044982	HES1	Q9D172	N/A	2.6	0.7
CB496534^(3)^	Ferritin	P09528	GO:0006955: immune response	2.3	0.1
CA057391^(2)^	Granulin	XP_537620.2	GO:0008283: cell proliferation	2.2	0.4
CA060176	Galectin like protein	O08573	GO:0006952: defense response	2.2	0.3
CA060021	Unknown		N/A	2.2	0.2
CA046740	Sequestosome 1; oxidative stress induced	NP_035148	GO:0007165: signal transduction	2.1	0.1
					
***Down regulated by rIFN-γ***					
					
CA057408	Annexin A2	P07356	GO:0050819 : negative regulation of coagulation	3.0	0.9
CB492780^(2)^	Id2 protein	P41136	GO:0045449: regulation of transcription	2.9	0.4
CA040487*	Ssa.17039		N/A	2.4	0.2
CK990422*	Ssa.2967		N/A	2.3	0.4
CA769647*	Ssa.3973		N/A	2.3	0.2
CB509992	18S ribosomal RNA gene, partial sequence	AY856868	N/A	2.1	0.1
CB491705	similar to PREDICTED: similar to ORF2	XP_425603.1	N/A	2.1	0.4
CB489453	Hypothetical protein 2	XP_417016	N/A	1.9	0.1
					
***Up regulated by rIL-1β***					
					
CA050149	IMMUNE-RESPONSIVE PROTEIN 1	XP_542615	N/A	27.7	10.4
CB511048	VHSV-induced C-lectin-like	AY572832	GO:0006955: immune response	23.2	10.8
CA061271	UNKNOWN		N/A	21.2	9.8
CK991068^(3)^	Hepcidin	AAO85553	GO:0006952: defense response	21.2	5.0
CA055080	Core histone macroH2A2.2; H2A histone family	AAH76893	GO:0044237: cellular metabolism	20.7	2.2
CA037550	60S ribosomal protein L30	P62889	GO:0006412: protein biosynthesis	19.0	8.5
CB496842^(3)^	C-type lectin	Q91ZW7	GO:0006955: immune response	18.4	4.1
CA054326	Tax1 binding protein 1	NP_006015.4	GO:0007165: signal transduction	15.9	10.5
CA063656	Cyclin L1	AY606031	N/A	14.2	10.4
CB511230	TAP2	AF002180	GO:0019882: antigen presentation	13.0	6.4
CK990725	RhoE	P61588	GO:0007165: signal transduction	12.5	5.6
CB504496^(3)^	Differentially regulated trout protein 1	AAG30030	GO:0006955: immune response	12.3	4.5
CA063738*	Nuclear factor of k light polypeptide gene enhancer (p49/p100)	NP_989744.	GO:0007165: signal transduction	12.2	2.9
CB498152*	Similar to MARCKS-like 1	NP_075385.1	GO:0005516: calmodulin binding	12.2	3.2
CK990269*	Connexin 31	NP_990262.1	GO:0007154: cell communication	12.1	2.4
CB516988*	PREDICTED: similar to heparan sulfate proteoglycan	XP_417362.1	GO:0006917: induction of apoptosis	12.1	4.4
CB512516*	Ssa.2559		N/A	12.1	9.0
CB516929*	Ssa.5205		N/A	12.0	4.7
CA060421^(2)^	CD83	AAP93912	GO:0006952: defense response	11.5	4.8
CB514313	Delta 1	Q61483	GO:0007154: cell communication	11.1	7.2
CA063097*	Ssa.6200		N/A	11.1	3.6
CB511158^(2)^	Precerebellin-like protein	AAF04305	GO:0006952: defense response	10.7	5.3
CA041210	MARCKS-like protein	P28667	GO:0005516: calmodulin binding	9.8	1.7
CA059788^(3)^	Inhibitor of nuclear factor kappa B alpha	AAH62524	GO:0006955: immune response	9.6	2.6
CA044647*	Ssa.3390		N/A	9.6	0.9
CA060755*^(2)^	Ssa.2872		N/A	9.4	2.3
CA051323*	Ssa.6734		N/A	9.3	5.0
CA053062^(3)^	5-aminolevulinic acid synthase	Q8VC19	GO:0009058: biosynthesis	8.3	2.6
CA061251	RAS-related C3 botulinum substrate 2	Q05144	GO:0006952: defense response	8.2	4.6
CB507662*	Ssa.383		N/A	8.1	0.4
CB501098*	Ssa.3941		N/A	7.9	4.4
CA058223	Guanylate binding protein 4	Q01514	GO:0006955: immune response	7.8	2.8
CB515854*	Similar to tumor necrosis factor, alpha-induced protein 2	NP_006282.2	GO:0006952: defense response	7.8	1.1
CA052773	Arginyl-tRNA synthetase	Q9D0I9	GO:0009058: biosynthesis	7.2	4.5
CA050427*^(3)^	Ssa9019		N/A	7.2	1.6
CB498855^(2)^	6-phosphofructo-2-kinase	P70265	GO:0044237: cellular metabolism	7.0	1.2
CA046850	DEAD (Asp-Glu-Ala-Asp) box polypeptide 5	P17844	GO:0016049: cell growth	6.8	3.7
CA056715^(3)^	Transcription factor JUN-B	P09450	GO:0045449: regulation of transcription	6.5	0.7
CA063635*	Ssa.5016		N/A	6.5	3.4
CB514104	Adipophilin (Adipose differentiation-related protein)	P43883	GO:0006810: transport	6.3	0.7
CA038364	Similar to ubiquinol-cytochrome c reductase complex	P00130	GO:0044237: cellular metabolism	6.2	1.6
CA054114*	Ssa.25362		N/A	6.2	1.6
CA770328	Tapasin	ABE27285	GO:0019882: antigen presentation	5.8	1.5
CA054633	ATP-binding cassette transporter 2	P21440	GO:0006810: transport	5.5	1.7
CB501435*	Omy.30821		N/A	5.4	0.6
CA055219^(2)^	CCAAT/enhancer binding protein beta,	AAH49401	GO:0045449: regulation of transcription	5.0	0.8
CA062875	Similar to IFN consensus sequence binding protein IRF-8	Q90871	GO:0045449: regulation of transcription	5.0	1.4
CB517430	Similar to epithelial stromal interaction 1	NP_150280.1	N/A	4.9	1.5
CA053490	Tumour necrosis factor receptor	CAD57165	GO:0007165: signal transduction	4.8	1.1
CK990939	Adipose differentiation-related protein	Q9MZE5	GO:0016020: membrane	4.7	0.5
CA051393^(2)^	Similar to interferon consensus sequence binding protein	NP_990747.1	GO:0045449: regulation of transcription	4.7	1.2
CB511966	Factor VIII	Q06194	GO:0006952: defense response	4.4	0.7
CA043801	Ssa.22345		N/A	4.4	1.2
CA062585	TAPBP protein...	Q8UUL4	GO:0019882: antigen presentation	4.4	0.9
CA053654*	Ssa.7103		N/A	4.3	0.9
CA060783	Similar to protein tyrosine phosphatase	NP_035336.1	GO:0006464: protein modification	4.2	1.1
CB515947	Ssa.13995		N/A	4.2	1.0
CB515614	Ssa.4774		N/A	4.1	1.2
CB497913	Omy.8396		N/A	3.9	0.4
CB498900	Ssa.14843		N/A	3.9	1.0
CA767815	sequestosome 1	NP_035148	GO:0007165: signal transduction	3.8	0.4
CA056501	Serine (or cysteine) proteinase inhibitor	Q07235	N/A	3.7	0.7
CA044867	Ssa.8481		N/A	3.7	1.0
CB494479	Transcription factor ETV6	P97360	GO:0045449: regulation of transcription	3.7	0.3
CA063565	Interferon regulatory factor 1	AAM77843	GO:0045449: regulation of transcription	3.6	0.7
CB496486^(2)^	Low molecular mass protein 7	P28063	GO:0006955: immune response	3.5	0.6
CB498525	Secretory granule proteoglycan core protein	XP_507828.1	N/A	3.5	0.7
CB515392	Stomatin	P54116	GO:0007165: signal transduction	3.5	0.2
CA052717	similar to Fc receptor-like 4	XP_547521.2	N/A	3.4	0.2
CA052500	TAP1A	Q9JJ59	GO:0019882: antigen presentation	3.4	0.2
CB494116	UNKNOWN		N/A	3.3	0.8
CB489126	Protein translation factor SUI1	Q9CXU9	GO:0006412: protein biosynthesis	3.2	0.3
CK990998^(3)^	similar to Protein translation factor SUI1 homolog	P48024	GO:0006412: protein biosynthesis	3.2	0.3
CA062698	Ssa.15825		N/A	3.2	0.4
CB499179	Leucine-rich-repeat protein	XM_039906	GO:0006952: defense response;	3.1	0.4
CK991328	Omy.3531		N/A	3.1	1.0
CA062450*	Similar to melanoma cell adhesion molecule	NP_075548.1	GO:0007155: cell adhesion	3.1	0.5
CA051372	Low molecular mass protein 2	Q60692	GO:0006955: immune response	3.0	0.9
CA057910*	Similar to programmed cell death 1 ligand 1	XP_424811.1	GO:0006955: immune response	3.0	0.4
CA056074	p60	AAH01874	GO:0007165: signal transduction	2.9	0.3
CB488180	ATP synthase	Q9DCX2	GO:0015986: ATP synthesis coupled proton transport	2.9	0.2
CA052868	NADPH oxidase cytosolic protein p40phox	AB192468	GO:0007165: signal transduction	2.9	0.3
CA043996*	Ssa.3435		N/A	2.8	0.3
CB503379*	Ssa.7962		N/A	2.8	0.2
CB511558	UNKNOWN		N/A	2.7	0.3
CA060176	Galectin like protein	O08573	GO:0006952: defense response	2.6	0.2
CB502697*	Transmembrane 4 superfamily member 3	NP_598210.1	N/A	2.6	0.3
CB498077^(4)^	Ferritin	P09528	GO:0006955: immune response	2.5	0.1
CA039745	Alpha-globin	CAA65955	GO:0006810: transport	2.5	0.2
CA062784	UNKNOWN		N/A	2.5	0.2
CB493123	Activating transcription factor 4,	Q6T3V3	GO:0045449: regulation of transcription	2.4	0.3
CB488782	Growth regulated nuclear 68 protein	Q61656	GO:0045449: regulation of transcription	2.3	0.1
					
***Down regulated by IL-1β***					
					
CA056515	UNKNOWN		N/A	5.4	2.8
CA041370	PREDICTED: similar to MAFB protein	XP_417353.1	GO:0045449: regulation of transcription	4.7	1.7
CA062828^(3)^	Id2 protein	P41136	GO:0045449: regulation of transcription	4.0	0.4
CB514524	xCDC46	BAA09949	GO:0045449: regulation of transcription	3.3	0.4
CB517923	Transforming protein myc	P01108	GO:0045449: regulation of transcription	3.1	0.7
CB502538	UNKNOWN		N/A	2.6	0.7
CA052685	Ribonucleotide reductase protein r2 class I	P11157	GO:0044237 : cellular metabolism	2.4	0.2

A melting curve for each PCR was determined by reading fluorescence every degree between 55°C and 95°C to ensure only a single product had been amplified. Two genes previously found to be useful for real time PCR in fish, elongation factor 1α and β-actin [91] were used as controls for normalization of expression. Neither of these genes were found to be altered in expression on the microarrays in these experiments.

To determine the relative expression level of candidate genes the method of Pfaffl [92] was used to obtain relative expression of candidate genes to both βactin and elongation factor 1α. Efficiency of amplification was determined for each primer pair using serial 10 fold dilutions of cDNA (1, 10, 100 & 1000 fold dilutions), performed on the same plate as the experimental samples. The efficiency was calculated as E = 10^(-1/s) ^where s is the slope generated from the serial dilutions, when Log dilution is plotted against ΔCT (threshold cycle number). For all real time PCRs triplicate reactions were performed.

Ratio=(Etarget)ΔCPTarget(control-sample)(Eref)ΔCPref(control-sample)
 MathType@MTEF@5@5@+=feaafiart1ev1aaatCvAUfKttLearuWrP9MDH5MBPbIqV92AaeXatLxBI9gBaebbnrfifHhDYfgasaacH8akY=wiFfYdH8Gipec8Eeeu0xXdbba9frFj0=OqFfea0dXdd9vqai=hGuQ8kuc9pgc9s8qqaq=dirpe0xb9q8qiLsFr0=vr0=vr0dc8meaabaqaciaacaGaaeqabaqabeGadaaakeaacqqGsbGucqqGHbqycqqG0baDcqqGPbqAcqqGVbWBcqGH9aqpdaWcaaqaaiabcIcaOiabbweafnaaBaaaleaacqqG0baDcqqGHbqycqqGYbGCcqqGNbWzcqqGLbqzcqqG0baDaeqaaOGaeiykaKYaaWbaaSqabeaacqqHuoarcqqGdbWqcqqGqbaudaWgaaadbaGaeeivaqLaeeyyaeMaeeOCaiNaee4zaCMaeeyzauMaeeiDaqhabeaaliabcIcaOiabbogaJjabb+gaVjabb6gaUjabbsha0jabbkhaYjabb+gaVjabbYgaSjabb2caTiabbohaZjabbggaHjabb2gaTjabbchaWjabbYgaSjabbwgaLjabcMcaPaaaaOqaaiabcIcaOiabbweafnaaBaaaleaacqqGYbGCcqqGLbqzcqqGMbGzaeqaaOGaeiykaKYaaWbaaSqabeaacqqHuoarcqqGdbWqcqqGqbaudaWgaaadbaGaeeOCaiNaeeyzauMaeeOzaygabeaaliabcIcaOiabbogaJjabb+gaVjabb6gaUjabbsha0jabbkhaYjabb+gaVjabbYgaSjabb2caTiabbohaZjabbggaHjabb2gaTjabbchaWjabbYgaSjabbwgaLjabcMcaPaaaaaaaaa@8393@

### Statistical analysis

For microarray studies standard errors of the mean are shown for each selected gene, where a cDNA is represented by more than a single feature the mean and SE for all features is presented. Data was also analysed by t-test within the Acuity software. For real time PCR, data was analysed using t-test for differences between samples and a Pearson correlation to confirm relationships. All micro array data were deposited to the Gene Expression Omnibus data bases [93] series **GSE5091 **using MIAME [94] guidelines.

## Authors' contributions

SAM performed microarray experiments, analysed the data, carried out the real time PCR and wrote the manuscript. JZ produced the recombinant cytokines, performed the cell stimulations and isolated the RNA. DFH and CJS were involved in the experimental design and drafting of the manuscript. All authors read and approved the final manuscript.
